# Preschool family irregularity and the development of sleep problems in childhood: a longitudinal study

**DOI:** 10.1111/jcpp.13060

**Published:** 2019-04-03

**Authors:** Maria Elisabeth Koopman‐Verhoeff, Fadila Serdarevic, Desana Kocevska, F. Fenne Bodrij, Viara R. Mileva‐Seitz, Irwin Reiss, Manon H.J. Hillegers, Henning Tiemeier, Charlotte A.M. Cecil, Frank C. Verhulst, Maartje P.C.M. Luijk

**Affiliations:** ^1^ Department of Child and Adolescent Psychiatry Erasmus University Medical Center–Sophia Children's Hospital Rotterdam The Netherlands; ^2^ The Generation R Study Group Erasmus Medical Center Rotterdam The Netherlands; ^3^ Department of Epidemiology Erasmus University Medical Center Rotterdam The Netherlands; ^4^ Institute of Education and Child Studies Leiden University Leiden The Netherlands; ^5^ Department of Pediatrics Erasmus University Medical Center–Sophia Children's Hospital Rotterdam The Netherlands; ^6^ Department of Social and Behavioral Science Harvard TH Chan School of Public Health Boston MA USA; ^7^ Institute of Psychiatry, Psychology and Neuroscience King's College London London UK; ^8^ Child and Adolescent Mental Health Centre Mental Health Services Capital Region, Research Unit Copenhagen University Hospital Copenhagen Denmark; ^9^ Department of Psychology, Education and Child Studies Erasmus University Rotterdam Rotterdam The Netherlands

**Keywords:** Family chaos, sleep duration, actigraphy, family routines, developmental psychopathology, longitudinal, accelerometer

## Abstract

**Background:**

Previous studies have shown that poor family environments are related to more sleep problems; however, little is known about how family irregularity in early life affects the development of sleep problems over childhood using objective sleep measures. The current study tests the hypothesis that early family irregularity contributes to the development of sleep problems.

**Methods:**

This population‐based study comprises 5,443 children from the Generation R Study. Family irregularity was measured with seven maternal‐reported questions on family routines when children were 2 and 4 years old. Mothers reported on sleep problems at child age 3, 6, and 10 years, whereas children completed questionnaires on sleep problems at age 10. Additionally, we used tri‐axial wrist accelerometers for five nights in 851 children (mean age 11.7 years) to assess sleep objectively.

**Results:**

Family irregularity was associated with more mother‐ and child‐reported sleep problems at ages 3, 6, and 10 years as well as with a shorter sleep duration and later objective sleep onset, but not with sleep efficiency or waking time. The association between family irregularity and multi‐informant subjective sleep problems at age 10 years was mediated by mother‐reported child psychopathology at age 6 years.

**Conclusions:**

Our findings show a long‐term robust association of preschool family irregularity with more sleep problems during childhood as well as shorter sleep duration and later sleep onset as measured objectively with actigraphy. In part, these sleep problems were associated with family irregularity by way of child psychopathology. These findings suggest that interventions improving preschool family irregularity, which are targeted to reduce child psychopathology, may also impact the development of sleep problems beneficially.

## Introduction

Sleep problems in children, such as difficulties falling asleep, nighttime awakenings, or nightmares (Gregory & Sadeh, 2016), are common complaints of parents and can disturb family life (O'Connor et al., 2007). Sleep problems frequently occur in general pediatric populations with prevalence estimates of up to 50% (Petit, Touchette, Tremblay, Boivin, & Montplaisir, 2007). The prevalence of childhood sleep problems typically declines with age, but in a subset of children sleep problems are persistent and predict poor outcomes later in life (Gregory & O'Connor, 2002). Despite the importance of sleep problems for later health and well‐being, questions about the etiology of sleep problems in school age children have yet to be fully addressed (Gregory & Sadeh, 2016). Previous research points to the importance of the family environment in relation to sleep; stressful family environments, a lack of parental rules, and family conflict have all been associated with sleep problems in children and adolescents (Adam, Snell, & Pendry, 2007; Gregory, Caspi, Moffitt, & Poulton, 2006). These negative family influences all occur more often in the context of an unpredictable family life (Gregory et al., 2006).

Family irregularity, that is, the lack of day‐to‐day family routines, refers to the lack of consistency in household routines, such as meal location and bedtime routines rather than more distal family influences (e.g. marital conflict, socioeconomic status) (Ivanova & Israel, 2005). The focus on daily routines also distinguishes family irregularity from perceived unpredictability of the home environment (like family chaos). Previous studies point at the importance of bedtime routines, which have been found to associate with longer sleep duration as measured with accelerometer in toddlers (Staples, Bates, & Petersen, 2015). Additionally, a recent review points to the potential of promoting bedtime routines as a feasible intervention for reducing sleep problems, especially in high‐risk families (Mindell & Williamson, 2018). However, the pathways linking the family irregularity and sleep problems are unclear. Higher levels of family irregularity hamper the ability of young children to develop a stable sleep onset and good quality sleep during the night (Billows et al., 2009; Buxton et al., 2015; Gregory, Eley, O'Connor, Rijsdijk, & Plomin, 2005; Spilsbury, Patel, Morris, Ehayaei, & Intille, 2017; Staples et al., 2015). Additionally, separate studies also find that family irregularity is associated with child psychopathology (Ivanova & Israel, 2006; Rijlaarsdam et al., 2016). While studies indicate that associations between sleep and child psychopathology are likely to be bidirectional (El‐Sheikh & Sadeh, 2015; Gregory & Sadeh, 2016), several studies show that specific symptoms sets of developmental psychopathology, such as ADHD or autism spectrum disorder, might be underlying sleep problems and not the reverse (Owens, 2005; Richdale & Schreck, 2009; Verhoeff et al., 2018). As such, child psychopathology may act as a mediator in the association between family irregularity and sleep problems.

Despite previous reports of an association between family irregularity and sleep problems, the literature is characterized by several gaps. First, studies have been primarily cross‐sectional; thus, it has not yet been possible to examine how family irregularity prospectively associates with the development of sleep problems in childhood. Second, studies to date have measured sleep exclusively using subjective reports; as such, the effects of family irregularity on objective indices of sleep have yet to be characterized. Third, no study to date has investigated whether child psychopathology mediates the association between family irregularity in early life and later sleep problems. Using data from a large population‐based study, we address these gaps by examining whether the lack of family routines is prospectively associated with sleep problems throughout childhood, using both parent‐ and child‐rated questionnaires as well as objective measures of sleep, and the different sleep assessments complement each other as they tap into different sleep domains (Sadeh, 2015). Moreover, we tested whether the association between family irregularity and child sleep problems is mediated by child psychopathology.

## Methods

### Participants

This study was embedded in Generation R, a prospective population‐based cohort from fetal life onward. All pregnant women (expected delivery date April 2002–January 2006) living in Rotterdam, the Netherlands, were invited to participate by their midwife or obstetrician during routine visits. All participants received questionnaires and were invited at the research center for observed behavioral assessment (previously described in detail (Kooijman et al., 2016)). The baseline participation rate was estimated at 61%. We obtained written informed consent from all participants and their parents. The Medical Ethical Committee of the Erasmus Medical Center Rotterdam approved the study.

Data on family irregularity at age 2 or 4 years were available for 5,842 children. Children without information on sleep problems on at least one assessment from age 3 years onward were excluded (*n *=* *399), yielding a sample size of 5,443 children for the present study (follow‐up rate 93.2%). In the analyses, the study population varies slightly due to missing data in different assessments rounds.

**Figure 1:**
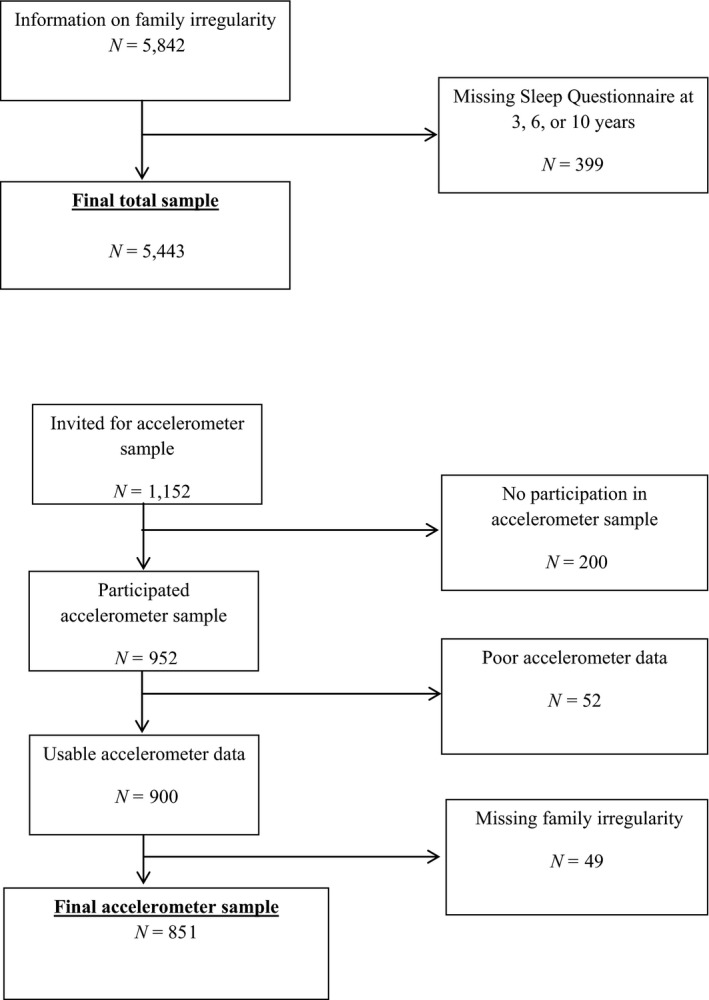
Flowchart: Family irregularity and sleep

A subsample of 1,153 children was recruited for an accelerometer sample by mail and phone. Of these, 953 children were willing to participate (response rate of 82%). Children without data on weekday sleep and those with corrupted measures were excluded. The final sample for the analyses with accelerometer measures consisted of 851 children. Mean age at the time of assessment was 11.7 (*SD* = 0.20) years (see Figure 1 for participant overview).

### Measures

#### Family irregularity

Family irregularity was a composite derived from seven questions about multiple domains of family irregularity reported by mothers when children were 2 and 4 years old. This family irregularity scale has previously been used in a reversed format as a measure of family regularity (Rijlaarsdam et al., 2016). The measure included two items on bedtime routines (i.e. ‘Do you have a set pattern or ritual with your child at bedtime?’ and ‘Has your child gone to bed in the evening at around the same time?’) at age 2 years. At age 4 years the measure included two questions on family meal location (i.e 'How often do you have breakfast/evening meal around the table together with your child/children?') and three questions on meal frequency (i.e. 'how often does your child eat breakfast/lunch/evening meals?'). Using confirmatory factor analysis (CFA), the seven irregularity items were combined into a single construct to represent family irregularity. CFA in Mplus version 7.11 was employed (Muthén & Muthén, 1998) to test the family irregularity measurement model using the weighted least squares means and variance adjusted (WLSMV) estimator. Model fit was established using the root mean squared error of approximation (RMSEA; acceptable fit ≤0.08), the comparative fit index and the Tucker–Lewis index (CFI and TLI; acceptable fit ≥0.90).

#### Child psychopathology

At age 6 years, the primary caregiver, mostly the mother, completed the Child Behavior Checklist for ages 1½–5 (CBCL/1½–5), a valid measure of child psychopathology (Achenbach & Rescorla, 2001). The CBCL/1½–5 is widely used internationally and has been found to be generalizable across 23 societies (Ivanova et al., 2010). Mothers rated various emotional and behavioral problems of the child in the previous 6 months on a three‐point scale (0 = not true, 1 = somewhat true, 2 = very true). All scores, except for five items referring to sleep, were combined into a Total Problems scale.

#### Mother‐reported child sleep problems

At age 1.5, 3, and 6 years, children's sleep problems were quantified with the Sleep Problems scale, one of the empirically derived scales of the CBCL/1½–5. The Sleep Problems scale comprises seven questions about sleep problems including items on dyssomnia (has trouble falling asleep; sleeps less than most children during the day and/or night; wakes up often during the night) and parasomnia (nightmares; talks or cries out in sleep). This scale is commonly used as a measure of sleep problems (Gregory & O'Connor, 2002). At age 10 years, we used the CBCL/6–18, which has a slightly different content to fit this older age range. The CBCL/6–18 does not have a specific Sleep Problems scale as the preschool version of the CBCL has. In order to keep the sleep measure consistent with the other two time points, we selected five sleep items from the CBCL/6–18 questionnaire to form a Sleep Problems scale. We used three questions representing dyssomnia symptoms: ‘Trouble with sleeping’; ‘Sleeps less than most kids’; and ‘Overtired with no good reason’, and two questions representing parasomnia symptoms: ‘Nightmares’ and ‘Talks or walks in sleep’ (internal consistency of *α = 0*.52), in line with a previous study (Verhoeff et al., 2018).

#### Child‐reported sleep problems

At age 10 years, dyssomnia symptoms were assessed by self‐report questionnaire asking six questions about their perceived sleep, that is, ‘Do you find it difficult to go to bed?’; ‘Do you find it difficult to fall asleep?’; ‘Do you think you get enough sleep?’; ‘If you wake up at night, do you find it difficult to fall asleep again?’; ‘Do you feel rested when you wake in the morning?’; and ‘When you come out of your bed in the morning, do you feel rested?’. These questions were derived from the widely used Sleep Disturbance Scale for Children (SDSC) (Bruni et al., 1996) and slightly rephrased for our pediatric population. There were three possible responses for each item: ‘No’, ‘Sometimes’, or ‘Yes’, which were scored on a three‐point Likert scale. Responses from all six items were summed to calculate a total score with an internal consistency of *α = 0*.64; higher scores indicate more sleep problems.

#### Objective sleep measures

At age 11 years, sleep was assessed with a tri‐axial wrist accelerometer (GENEActiv; Activinsights, UK); the children wore the devices for nine subsequent days on their nondominant wrist, including five school days and four weekend days. This measure has been validated in children and in adults. In children, thresholds have been calculated to assess sedentary behavior (sitting/lying) with a sensitivity of 97–98% and a specificity of 74–78% (Hildebrand, Hansen, van Hees, & Ekelund, 2017) and the GENEActive has been found to correlate well with both sleep diaries and self‐reported sleep duration (Nascimento‐Ferreira et al., 2016). In adults, it is shown to be a valid measure of sleep, which provides comparable estimates to other accelerometer brands (Rosenberger, Buman, Haskell, McConnell, & Carstensen, 2016). Additionally, children filled out a sleep diary each morning answering questions about their sleep during the previous night. The questions ‘What time did you go to bed?’ and ‘What time did you wake up?’ were used as indicators in the actigraphy analysis to guide the accelerometer‐based sleep onset and waking detection. The GENEActiv accelerometers record raw accelerometer data, and for the current study, they were set a frequency of 50 Hz. The binary files were processed with the R‐package GGIR (van Hees et al., 2014). The processing included auto calibration with gravity as reference, detection of atypical values and nonwear, and calculation of the average acceleration. Nights were excluded if the wear time was under 6 hr or if sleep time was calculated as being less than 4 hr. This procedure generated the following variables: sleep duration, sleep efficiency, sleep onset, and waking time (van Hees et al., 2015). Sleep duration is the total time asleep during the night, indicating the time between falling asleep and waking minus the time scored as awake. Sleep efficiency is the total sleeping time divided by bed time and waking time and is presented as a percentage. Sleep onset is the time a child fell asleep, and waking time is the time children woke in the morning. For sake of homogeneity, for the measures of sleep duration, sleep efficiency, sleep onset, and sleep waking time in the current study only school days were included in the analyses, representing the typical pattern of weekday sleep to minimize the influence of atypical weekend events. We did, however, integrate weekend sleep in a sensitivity analysis to test the robustness of associations. For more information please refer to Koopman‐Verhoeff et al., 2018.

### Confounders

Based on the literature (Billows et al., 2009; Gregory et al., 2005), the following variables were considered possible confounders in the association between family irregularity and sleep. Sex of the child was obtained from the medical records completed by community midwives and obstetricians, and information on other maternal and child characteristics was obtained by questionnaires. Child ethnicity was based on country of birth of the parents, coded as, Dutch, Other‐Western, and non‐Western. Additionally, maternal education was defined by the highest attained educational level and classified into three categories (low, middle, and high education). Prenatal maternal psychopathology was assessed using the Brief Symptom Inventory (BSI, De Beurs, 2004). We considered other potential confounding factors such as siblings, bed‐sharing, and asthma but did not add them to our final models. While bed‐sharing at age 2 years was found to be positively correlated with sleep duration at age 11 years (indicating a longer sleep duration) and negatively correlated with preschool family irregularity (indicating less family irregularity), it did not affect results once included as an additional confounder. The other factors, such as siblings and medical data such as asthma, were not confounders since they were unrelated to the exposure.

### Statistical analysis

For ease of comparison over the different instruments and time points, we standardized all independent and dependent variables. For each of the steps described below, we constructed two models. The first model was adjusted for child sex and child age at sleep assessment. In the second model, the following confounders were included: child sex, child age at sleep assessment, gestational age, ethnicity, maternal age at birth, maternal psychopathology, and maternal educational level. Additionally, we controlled for previous sleep problems reported by the mother at age 1.5 years. The analyses were performed in four steps.

First, we examined the *longitudinal* association between family irregularity and mother‐reported sleep problems measured at ages 3, 6, and 10 years. To this end, we used generalized linear mixed models (GLMM) and estimated associations using standardized beta coefficients and 95% confidence intervals (CI). This analysis was conducted exclusively with mother‐reported sleep problems given the availability of repeated measures. All models included a subject‐level random intercept and slope to account for repeated measures of child sleep problems and to model child‐specific variable effect. GLMM are robust to loss to follow‐up under the missing at random assumption.

Second, we used linear regression models to derive individual estimates of the prospective association of preschool family irregularity with *multi‐informant* sleep problems, including (a) maternal‐reported (age 3, 6, and 10) and (b) child‐reported (age 10) sleep. Third, we tested associations between family irregularity and objective measures of sleep (age 11), in the subsample with available accelerometer data using linear regression models. Fourth, we tested whether child psychopathology at age 6 years *mediated* the association between family irregularity and multi‐informant sleep problems at age 10 years. We also tested whether child psychopathology acted as a mediator between family irregularity and objective measures of sleep. We ran mediation models with 99% bias‐corrected bootstrap confidence intervals applying 5,000 bootstrap samples using the PROCESS macro in SPSS (Hayes, 2015).

### Sensitivity analyses

In addition to the main analyses described above, we ran several sensitivity analyses to test the robustness of our findings. First, to minimize the content overlap between some of the items in the family irregularity and sleep, we reran the CFA analysis to extract a factor of family irregularity without the bed time routine‐related items (i.e. including only the five items on mealtime location and mealtime routines). We reran all models using the adapted version of the family irregularity construct. Second, in the accelerometer sample, all models concerning objective sleep measures were rerun using combined weekend and weekday sleep. To reduce bias associated with missing data, we used multiple imputations for missing values of the confounders. Ten imputed datasets were created and analyzed separately after which the results were pooled. The statistical analyses were performed using the SPSS version 22.0 for Windows (IBM Corp., Armonk, NY, USA), MPlus version 7.11 (Muthén & Muthén, Los Angeles, CA, USA) using Monte Carlo integration techniques and maximum likelihood estimation with robust standard errors. Longitudinal analysis was performed using SAS version 9.3 (SAS Institute Inc., Cary, NC).

## Results

Characteristics of the children in the total sample and the accelerometer sample are presented in Table 1. Children in the accelerometer sample had an average sleep duration of 7 hr and 45 min (*SD* = 42 min), a mean sleep efficiency of 81% (*SD* = 5.5%), a mean sleep onset time at 22:04 (*SD* = 55 min), and a mean waking time at 6:46 (*SD* = 58 min). Table S1 shows the correlations among the sleep variables, showing very modest correlations between the sleep problem scales and accelerometer measures of sleep duration and patterns; for example, sleep problems reported by the mother at age 6 years are negatively correlated with sleep duration (*r = −*.117).

**Table 1:** Characteristics of the study population

	*N*	Total sample *N *=* *5,443	*N*	Accelerometer sample *N *=* *852
Child characteristics				
Sex (% girls)	5,443	50.1	852	52.3
Gestational age at birth (weeks)	5,418	39.84 (1.80)	852	39.65 (2.24)
Ethnicity				
Dutch%	3,518	64.6	715	84.0
Other‐Western%	509	9.4	49	5.8
Non‐Western%	1,416	26.0	88	10.3
Sleep duration (hours:minutes)		–	852	7.45 (0.42)
Sleep efficiency (%)		–	852	84 (5.1)
Sleep onset (time to fall asleep)		–	852	22.04 (0.55)
Sleep problem score (maternal report)				
At 3 years	4,695	1.91 (2.12)	778	1.68 (1.91)
At 6 years	4,805	1.33 (1.81)	808	1.12 (1.62)
At 10 years	3,960	0.83 (1.22)	793	0.83 (1.23)
Sleep problem score at 10 years (child report)	3,598	10.88 (2.47)	772	11.00 (2.47)
Family irregularity	5,443	1.40 (0.39)	852	1.32 (.34)
Maternal characteristics				
Age at inclusion (years)	5,442	31.40 (4.66)	852	32.33 (3.85)
Educational level				
No education/primary school%	358	6.6	15	1.8
High school/lower vocational training%	2,054	37.7	277	32.5
Higher vocational or academic training%	3,031	55.7	560	65.7
Psychopathology score	5,443	0.24 (0.31)	852	0.19 (0.24)

Data represent means (*SD*s) unless specified otherwise.

### Mother‐reported sleep problems

The results of the GLMM model indicate a longitudinal association between preschool family irregularity and mother‐rated sleep problems. While this association was found to decline over time, it was still observable at age 10 years (age 3, *β* =0.21, 95%CI: 0.17 to 0.25; age 6, *β* =0.16, 95%CI: 0.12 to 0.20; age 10, *β* =0.10, 95%CI: 0.06 to 0.14). To illustrate, Table 2 shows the associations of preschool family irregularity with sleep problems at ages 3, 6, and 10 years based on the linear regression models, adjusted for confounders (*β* =0.13, 95%CI: 0.10 to 0.16, *p *<* *.01; *β* =0.11, 95%CI: 0.08 to 0.14, *p *<* *.01; *β* =0.06, 95%CI: 0.02 to 0.10, *p *<* *.01, respectively). Results slightly attenuated after additionally controlling for previous sleep problems at age 1.5 years. In Figure S1, we show associations between family irregularity and mother‐rated sleep problems at all ages (adjusted for confounders and also additionally for previous sleep problems).

**Table 2:** The association between preschool family irregularity and mother‐ and child‐reported sleep problems at ages 3, 6, and 10 years (total sample)

Family irregularity	Maternal reported									Child reported		
	3 year *N *=* *4,695			6 year *N *=* *4,805			10 year *N *=* *3,960			10 year *N *=* *3,598		
	*β*	CI	*p*	*β*	CI	*p*	*β*	CI	*p*	*β*	CI	*p*
Model 1	.18	.15 to .21	<.01	.17	.14 to .20	<.01	.06	.03 to .10	<.01	.06	.03 to .10	<.01
Model 2	.13	.10 to .16	<.01	.11	.08 to .14	<.01	.06	.02 to .10	<.01	.08	.04 to .13	<.01
Model 3	.10	.07 to .13	<.01	.08	.05 to .11	<.01	.05	.01 to .09	<.01	.08	.04 to .12	<.01

Model 1 was unadjusted. Model 2 was sex, age of the child at sleep assessment, child's gestational age, and child's ethnicity, maternal age at birth, maternal education, and maternal psychopathology. Model 3 was adjusted for previous baseline sleep problems at age 1.5 years.

### Child‐reported sleep problems

Preschool family irregularity was prospectively associated with higher levels of child‐reported sleep problems at age 10, over and above adjustment for confounders (*β* =0.08, 95%CI: 0.04 to 0.13, *p *<* *.01). Results remained similar after additionally controlling for previous sleep problems at age 1.5 years.

### Objective sleep

Family irregularity in the preschool period was prospectively associated with sleep duration and sleep onset at age 11. Higher levels of family irregularity were associated with a shorter sleep duration (*β = −*0.09, 95%CI: −0.16 to −0.01, *p *=* *.02) and a later sleep onset (*β* =0.10, 95%CI: 0.03 to 0.17, *p *<* *.01). Family irregularity was not associated with the other objective sleep parameters (i.e. sleep efficiency and waking time; Table 3). Results remained similar after additionally controlling for previous sleep problems at age 1.5 years.

**Table 3:** Associations between preschool family irregularity and objective schooldays sleep at age 11 years (accelerometer sample)

Family irregularity	Sleep duration *N *=* *865			Sleep efficiency *N *=* *865			Sleep onset *N *=* *865			Wake time *N *=* *865		
	*β*	CI	*p*	*β*	CI	*p*	*β*	CI	*p*	*β*	CI	*p*
Model 1	−.10	−.17 to −.03	<.01	.01	−.06 to .08	.80	.11	.04 to .18	<.01	.02	−.05 to .09	.55
Model 2	−.09	−.16 to −.01	.02	.02	−.05 to .10	.56	.10	.03 to .17	<.01	.01	−.06 to .08	.75
Model 3	−.08	−.16 to −.01	.02	.02	−.05 to .10	.55	.10	.03 to .17	<.01	.01	−.06 to .08	.79

Model 1 was unadjusted. Model 2 was adjusted for sex, age of the child at sleep assessment, child's gestational age, and child's ethnicity, maternal age at birth, maternal education, and maternal psychopathology. Model 3 was adjusted for previous baseline sleep problems at age 1.5 years.

### Child psychopathology as a mediator

Child psychopathology was tested as a potential mediator between preschool family irregularity and sleep problems. With bootstrapped mediation models, we demonstrate an indirect effect of family irregularity on child sleep problems (full‐mediation for mother report and partial mediation for child report at age 10 years) via child psychopathology at age 6 years (*mother‐reported sleep problems:* adjusted *β*: 0.14; 95%CI: 0.04 to 0.33 for [Ratio of indirect to direct effect 40%]; *child‐reported sleep problems:* adjusted beta: 0.05; 95%CI: 0.00 to 0.16 [Ratio of indirect to direct effect 13%]). Child psychopathology did not mediate the association between family irregularity and objective sleep indices.

### Sensitivity analyses

For the sensitivity analyses, we reran all models using an adapted version of the preschool family irregularity construct without the items on bedtime routines. Findings were generally robust, except for the association between family irregularity and mother‐reported sleep problems at age 10 years, which was attenuated (Tables S2 and S3). Table S4 shows that the findings remained consistent when we reran the models with the objective sleep measures combining weekend plus weekday sleep.

## Discussion

This study is unique as it demonstrated a longitudinal association of family irregularity with repeatedly measured, multi‐informant sleep problems, as well as objective measures of sleep, even when controlled for previous sleep problems at age 1.5 years. We highlight three key findings here. First, we found that family irregularity experienced by toddlers is associated with long‐term mother‐reported and child‐reported sleep problems and shorter sleep duration and a later sleep onset. These findings are robust across time, raters, and methods of assessment (reported and actigraphy), pointing to family irregularity as an early risk marker for later sleep problems. The differential effects for children at various ages and the decline of the effect between family irregularity and sleep problems over time may represent a regression dilution effect. Second, sensitivity analyses indicated that the effect was not purely driven by family regularity items related to bedtime routines. Third, child psychopathology at age 6 mediated the association between family irregularity and reported sleep problems, but did not influence the relation with objective sleep parameters.

By using objective sleep measures, we complement and extend the findings of the only other existing study to examine family irregularity in relation to child sleep, which showed that family irregularity is cross‐sectionally related to child‐reported shorter sleep duration and delayed sleep onset (Billows et al., 2009). A potential mechanism for this association is that family irregularity interferes with cues that can act as Zeitgebers (Ehlers, Frank, & Kupfer, 1988). Zeitgebers are external signals that help individuals to entrain a day–night rhythm in concordance with the 24‐hr light–dark cycle of the earth. In daily life, these cues can help children to get ready to go to bed. Children raised under irregular family circumstances might lack those cues, or might receive irregular cues and struggle to adequately adapt their circadian rhythms. Moreover, adolescents with a delayed sleep onset often have chronic insufficient sleep (Billows et al., 2009; Carskadon, Acebo, & Jenni, 2004; Tarokh, Saletin, & Carskadon, 2016). Importantly, the current findings underscore the potential of family interventions targeted at family irregularity, a documented modifiable risk factor. Indeed, a previous randomized trial targeting household routines aiming to reduce obesity in preschool children effectively increased sleep duration (Haines et al., 2013). In contrast, other family circumstances which influence sleep are harder to address, such as family conflict and maltreatment. Potentially, the intervention targeting family irregularity can be extended to increase sleep duration throughout childhood. As such, it will be important to examine the association between family irregularity and objective sleep in adolescence in future research. We were able to identify a risk factor for child sleep problems that is known to be modifiable.

Psychopathology was investigated as a potential pathway linking family irregularity and sleep problems, given that both have been previously associated with child psychopathology (Gregory & Sadeh, 2016; Ivanova & Israel, 2006). The association between family irregularity and mother‐reported sleep problems at age 10 years was fully mediated by child psychopathology. This mediation may reflect shared method variance due to the use of maternal reports for the determinant, mediator, and outcome; however, partial mediation was observed when using child‐rated sleep problems as the outcome. Overall, these findings suggest long‐term associations between early family irregularity, child psychopathology, and child subjective sleep problems.

The association between family irregularity and psychopathology is well known (Ivanova & Israel, 2006), as is the association between sleep problems and psychopathology (Gregory & Sadeh, 2016). Previous studies have suggested that sleep problems result from psychopathology and not the other way around (Owens, 2005; Richdale & Schreck, 2009; Verhoeff et al., 2018). However, it is also possible that sleep problems lead to psychopathology or that the associations are bidirectional (El‐Sheikh & Sadeh, 2015; Gregory & Sadeh, 2016). While our study lends support to the psychopathology as a mediator between early family irregularity and later sleep problems, we are unable to rule out alternative pathways and it is therefore premature to conclude about the direction of the relations between sleep, family circumstances, and psychopathology. Future longitudinal studies with repeated measures of these variables are needed to disentangle directionality.

In contrast, child psychopathology did not mediate the association of preschool family irregularity and objective sleep. These objective and subjective measures reflected not only different assessments but different, albeit related, outcomes. The sleep items in the CBCL tap on experiences of dyssomnia and parasomnia symptoms, whereas accelerometer data index parameters such as sleep duration, efficiency, sleep onset time, and waking time. These differences were indeed evidenced by the small correlation between the sleep problem scales and accelerometer measures in our sample, consistent with prior reports (Gregory et al., 2011). As such, our data suggested that child psychopathology may play a stronger role in the development of dyssomnias and parasomnias, as opposed to alterations in accelerometer sleep patterns.

### Limitations and strengths

The findings of the current study should be considered in light of some limitations. First, in the current study we used maternal reports of family irregularity – it would have been optimal to use objective measures of family irregularity. However, objective measures of family irregularity are often situation and time dependent and not feasible. Second, we did not have repeated measures of family irregularity. This precludes the possibility to examine how changes in family irregularity over time relate to changes in sleep problems. Third, because of the population‐based nature of the current study, the generalizability to clinical samples will need to be established in future. This study had, however, also multiple strengths. First, we made use of accelerometer measures of sleep, which is a reliable measure of sleep duration, efficiency, sleep onset time, and waking time. Second, we obtained questionnaire sleep measures across multiple raters and at multiple time points, which enabled us to study the course of sleep problems over time. Third, because of our design and large sample size, we were able to control for multiple confounders.

## Conclusion

In summary, this population‐based study supports the important role of early family irregularity, over and above the role of bedtime routines, in shaping the development of sleep across childhood. Preschool family irregularity in toddlers can have lasting consequences on subjectively assessed sleep problems up to age 10 years, such as dyssomnias and parasomnias, as well as shorter sleep duration and delayed sleep onset, based on accelerometer data. This study also points to child psychopathology as a potential pathway linking family irregularity in early life and later sleep problems. These robust, long‐term findings suggest that interventions targeting preschool family irregularity might be potential avenues for reducing both the development of sleep problems and the risk of child psychopathology.

## Ethical considerations

The Medical Ethical Committee of the Erasmus Medical Center Rotterdam approved the study. The authors obtained written informed consent from all participants and their parents.

**Key points**

- Little is known about the role of preschool family irregularity in the development of, in particular, objectively measured sleep problems.
- In this general population study, preschool family irregularity was prospectively associated with more subjective sleep problems, as rated by both mothers and children, and predicted shorter sleep duration and a later objective sleep onset.
- This study is unique in finding a robust, long‐term association of preschool family irregularity and sleep problems and sleep patterns, over and above the previously established influence of bedtime routines on child sleep.
- Our study identified a risk factor, family irregularity, for child sleep problems that is known to be feasible and relatively easily modifiable.

## Supporting information


**Table S1.** Correlation between sleep problem scales and accelerometer measures.
**Table S2.** Association between preschool family irregularity (without bedtime routines) and mother‐ and child‐reported sleep problems at ages 3, 6, and 10 years (total sample).
**Table S3.** Associations between preschool family irregularity (without bedtime routines) at age 4 years and objective sleep at age 11 years (accelerometer sample).
**Table S4.** Associations between preschool family irregularity at age 2 and 4 years and objective sleep at age 11 years (accelerometer sample) (weekdays and weekend days combined).
**Figure S1.** The longitudinal association of family irregularity and sleep problems.
