# Supplementary material for: Preschool family irregularity and the development of sleep problems in childhood: a longitudinal study
Source: J Child Psychol Psychiatry. 2019 Apr 3;60(8):857–65. doi: 10.1111/jcpp.13060 (PMC6850317; doi:10.1111/jcpp.13060)
Supplement: Supplementary file 1 — Table S1. Correlation between sleep problem scales and accelerometer measures. Table S2. Association between preschool family irregularity (without bedtime routines) and mother‐ and child‐reported sleep problems at ages 3, 6, and 10 years (total sample). Table S3. Associations between preschool family irregularity (without bedtime routines) at age 4 years and objective sleep at age 11 years (accelerometer sample). Table S4. Associations between preschool family irregularity at age 2 and 4 years and objective sleep at age 11 years (accelerometer sample) (weekdays and weekend days combined). Figure S1. The longitudinal association of family irregularity and sleep problems. [file JCPP-60-857-s001.docx]

**Supporting information – Pre-school family irregularity and the development of sleep problems in childhood: a longitudinal study – by Luijk et al.**

**Table S1**. Correlation between sleep problem scales and accelerometer measures.

|  | 1 | 2 | 3 | 4 | 5 | 6 | 7 | 8 | 9 |
| --- | --- | --- | --- | --- | --- | --- | --- | --- | --- |
| 1. Sleep duration, accelerometer | 1 |  |  |  |  |  |  |  |  |
| 2. Sleep efficiency, accelerometer | 0.521** | 1 |  |  |  |  |  |  |  |
| 3. Sleep onset, accelerometer | -0.648** | 0.060 | 1 |  |  |  |  |  |  |
| 4. Waking time, accelerometer | -0.062 | -0.127** | 0.581** | 1 |  |  |  |  |  |
| 5. Sleep problems 1.5 years, maternal report | -0.086* | -0.053 | 0.078* | 0.053 | 1 |  |  |  |  |
| 6. Sleep problems 3 years, maternal report | -0.066 | -0.047 | 0.028 | -0.013 | 0.402** | 1 |  |  |  |
| 7. Sleep problems 6 years, maternal report | -0.117** | -0.052 | 0.069* | -0.019 | 0.350** | 0.439** | 1 |  |  |
| 8. Sleep problems 10 years, maternal report | -0.040 | 0.026 | 0.017 | -0.061 | 0.060 | 0.179** | 0.326** | 1 |  |
| 9. Sleep problems 10 years, child report | -0.049 | -0.013 | 0.058 | 0.033 | -0.016 | 0.053 | 0.096** | 0.213** | 1 |

Pearson’s r coefficients are reported for correlations with the sleep parameters.
* Statistically significant at *p*<0.05
** statistically significant at *p*<0.01

| **Table S2.** Association between pre-school family irregularity (without bedtime routines) and mother- and child-reported sleep problems at ages 3, 6, and 10 years (total sample). | | | | | | | | | | | | | | | |
| --- | --- | --- | --- | --- | --- | --- | --- | --- | --- | --- | --- | --- | --- | --- | --- |
|  | Maternal reported | | | | | | | | | | |  | Child  reported | | |
|  | 3 year N = 4695 | | |  | 6 year N = 4805 | | |  | 10 year N = 3960 | | |  | 10 year N = 3598 | | |
| Family irregularity | β | *CI* | *p* |  | β | *CI* | *p* |  | β | *CI* | *p* |  | β | *CI* | *p* |
| Model 1 | .14 | .11–.17 | <.01 |  | .13 | .10–.16 | <.01 |  | .02 | -.01–.06 | .24 |  | .05 | .01–.09 | <.01 |
| Model 2 | .09 | .05–.12 | <.01 |  | .07 | .05–.10 | <.01 |  | .01 | -.02–.05 | .51 |  | .06 | .02–.10 | <.01 |
| Model 3 | .06 | .03–.09 | <.01 |  | .07 | .04–.10 | <.01 |  | .02 | -.02–.06 | .56 |  | .06 | .02–.10 | <.01 |

Model 1 was unadjusted. Model 2 was sex, age of the child at sleep assessment, child’s gestational age, and child’s ethnicity, maternal age at birth, maternal education, and maternal psychopathology. Model 3 was adjusted for previous baseline sleep problems at age 1.5 years.

| **Table S3.** Associations between pre-school family irregularity (without bedtime routines) at age 4 years and objective sleep at age 11 years (accelerometer sample). | | | | | | | | | | | | | | | |
| --- | --- | --- | --- | --- | --- | --- | --- | --- | --- | --- | --- | --- | --- | --- | --- |
|  | Sleep duration  N = 865 | | |  | Sleep efficiency  N = 865 | | |  | Sleep onset  N = 865 | | |  | Wake time  N = 865 | | |
| Family irregularity | β | *CI* | *p* |  | β | *CI* | *p* |  | β | *CI* | *p* |  | β | *CI* | *p* |
| Model 1 | -.12 | -.21– -.03 | <.01 |  | .02 | -.07–.11 | .67 |  | .15 | .06–.24 | <.01 |  | .05 | -.04-.13 | .30 |
| Model 2 | -.10 | -.19– -.01 | .03 |  | .02 | -.07–.12 | .59 |  | .13 | .04–.21 | <.01 |  | .04 | -.05–.12 | .39 |
| Model 3 | -.10 | -.19–-.01 | .03 |  | .03 | -.07–.12 | .57 |  | .13 | .04–.21 | <.01 |  | .04 | -.05–.12 | .41 |

Model 1 was unadjusted. Model 2 was sex, age of the child at sleep assessment, child’s gestational age, and child’s ethnicity, maternal age at birth, maternal education, and maternal psychopathology. Model 3 was adjusted for previous baseline sleep problems at age 1.5 years.

| **Table S4.** Associations between pre-school family irregularity at age 2 and 4 years and objective sleep at age 11 years (accelerometer sample) (weekdays and weekend days combined). | | | | | | | | | | | | | | | |
| --- | --- | --- | --- | --- | --- | --- | --- | --- | --- | --- | --- | --- | --- | --- | --- |
|  | Sleep duration  N = 865 | | |  | Sleep efficiency  N = 865 | | |  | Sleep onset  N = 865 | | |  | Wake time  N = 865 | | |
| Family irregularity | β | *CI* | *p* |  | β | *CI* | *p* |  | β | *CI* | *p* |  | β | *CI* | *p* |
| Model 1 | -.08 | -.15–-.01 | <.05 |  | .04 | -.03–.12 | .24 |  | .13 | .06–.19 | <.01 |  | .05 | -.01–.11 | .44 |
| Model 2 | -.06 | -.14–.01 | .07 |  | .05 | -.02–.13 | .17 |  | .11 | .05–.18 | <.01 |  | .04 | -.02–.10 | .20 |
| Model 3 | -.06 | -.13–.01 | .08 |  | .05 | -.02–.13 | .16 |  | .11 | .04–.17 | <.01 |  | .04 | -.02–.10 | .21 |

Model 1 was unadjusted. Model 2 was sex, age of the child at sleep assessment, child’s gestational age, and child’s ethnicity, maternal age at birth, maternal education, and maternal psychopathology. Model 3 was adjusted for previous baseline sleep problems at age 1.5 years.

**Figure S1.** The longitudinal association of family irregularity and sleep problems.

**A**

**B**

**A.** The associations of pre-school family irregularity and sleep problems for each age category were based on linear regression analysis of family irregularity and sleep problems per each age category, adjusted for gender, age of the child at sleep assessment, child’s gestational age, and child’s ethnicity, maternal age at birth, maternal education, and maternal psychopathology. Betas are averaged from 10 imputed data sets.
**B.** The associations of pre-school family irregularity and sleep problems for each age category were based on generalized linear mixed models of family irregularity and sleep problems per age category. Additionally, they were adjusted for gender, age of the child at sleep assessment, child’s gestational age, and child’s ethnicity, maternal age at birth, maternal education, and maternal psychopathology. Betas are averaged from 10 imputed data sets.
